# Increased risk for development of coronary artery calcification in subjects with non-alcoholic fatty liver disease and systemic inflammation

**DOI:** 10.1371/journal.pone.0180118

**Published:** 2017-07-07

**Authors:** Jihyun Kim, Da Young Lee, Se Eun Park, Cheol-Young Park, Won-Young Lee, Ki-Won Oh, Sung-Woo Park, Eun-Jung Rhee

**Affiliations:** Department of Endocrinology and Metabolism, Kangbuk Samsung Hospital, Sungkyunkwan University School of Medicine, Seoul, Korea; Medical University Innsbruck, AUSTRIA

## Abstract

**Background:**

Recent studies have suggested the importance of non-alcoholic fatty liver disease (NAFLD) and systemic inflammation in the development of atherosclerosis. The aim of this study was to compare the risk for coronary artery calcification (CAC) development according to the status of NAFLD and inflammation over four years of follow-up in subjects without baseline CAC.

**Methods:**

A total of 1,575 participants in a health screening program were divided into four groups according to baseline NAFLD state and high-sensitivity C-reactive protein (hs-CRP) (median 0.06 mg/L) levels as follows: no NAFLD and hs-CRP <0.06 mg/L, no NAFLD and hs-CRP ≥0.06 mg/L, NAFLD and hs-CRP <0.06 mg/L, and NAFLD and hs-CRP ≥0.06 mg/L. Coronary artery calcium score (CACS) was repeatedly measured by multi-detector computed tomography at four-year intervals and CAC development during those intervals was monitored in subjects with baseline CACS = 0.

**Results:**

Over four years, 148 subjects (9.4%) developed CAC. The proportion of subjects who developed CAC was significantly higher in subjects with NAFLD at baseline compared with those without NAFLD at baseline (6.8 vs. 12.4%, p<0.01), and it was also higher in subjects with hs-CRP ≥0.06 mg/L compared with those with hs-CRP <0.06 mg/L (7.2 vs. 11.5%, p<0.01). In addition, the proportion of subjects who developed CAC was highest in subjects with NAFLD and hs-CRP ≥0.06 mg/dL, followed by subjects with NAFLD, subjects without NAFLD and hs-CRP ≥0.06 mg/L, and subjects without NALFD and hs-CRP <0.05 mg/L at baseline, in that order (13.7, 10.0, 8.3, and 5.8%, respectively; p for trend<0.01). The odds ratio for CAC development was highest in subjects with NAFLD and hs-CRP ≥0.06 mg/L (1.67, 95% CI 1.01–2.77), though it was attenuated after adjustment for body mass index.

**Conclusions:**

The concomitant presence of NAFLD and systemic inflammation as assessed by hs-CRP increases the risk of CAC development over four years.

## Introduction

Non-alcoholic fatty liver disease (NAFLD) is defined as a condition of fat deposition in the liver that comprises over 5% of the total liver weight in individuals without known viral or other liver diseases, and without a history of excessive daily alcohol consumption (defined as a daily intake of over 20 g for females and 30 g for males) [1,2]. NAFLD is the most common chronic liver disease in Western countries and is closely related to insulin resistance and oxidative stress. The prevalence of NAFLD is on the rise in Korea, and 18–47% of the participants in the medical health check-up program have been reported to have NAFLD [3–5].

The association of NAFLD with obesity may lead to the connection between NAFLD and the development and progression of cardiovascular disease (CVD). Nonalcoholic fatty liver disease itself may promote CVD by contributing to insulin resistance and atherogenic dyslipidemia, which are important risk factors for cardiovascular disease [6,7]. Increased release of chemical messengers from visceral adipose tissue, including inflammatory cytokines, leads to adipose tissue inflammation that aggravates insulin resistance and facilitates atherosclerosis development and cardiovascular disease [7,8]. However, Kolak et al. [9] observed adipose tissue inflammation in individuals with NAFLD independent of obesity, suggesting that the release of mediators from NAFLD causes adipose tissue inflammation, and therefore systemic inflammation and CVD. Accordingly, systemic inflammation, which is reflected by surrogate markers such as high-sensitivity C-reactive protein (hs-CRP), has been proposed as a key factor in the relationship between NAFLD and CVD.

Coronary artery calcium score (CACS) is a non-invasive and useful surrogate marker that effectively reflects atherosclerotic burden in subjects with or without CVD [10]. Previous studies have shown that NAFLD is independently associated with the prevalence of coronary artery calcification (CAC) [11–16]. On the other hand, other studies have reported conflicting results, arguing that the association of NAFLD and CAC is an obesity-related epiphenomenon [17].

To date, no studies have investigated CAC development in subjects with NAFLD according to the level of systemic inflammation. Thus, we sought to examine whether the degree of systemic inflammation assessed by hs-CRP in patients with NAFLD could affect CAC development in subjects without baseline CAC over four years of follow-up.

## Methods

### Study population

This was a longitudinal, retrospective sub-study of the Kangbuk Samsung Health Study, which included participants in a medical health checkup program at the Health Promotion Center of Kangbuk Samsung Hospital, Sungkyunkwan University, Seoul, Korea. The purpose of the medical health checkup program is to promote the health of employees through regular health checkups and to facilitate early detection of existing diseases. Most of the examinees are employees or family members of various industrial companies from around the country. The costs of the medical examinations are largely paid for by the employers, and a considerable proportion of the participants undergo examinations annually or biannually.

The initial study population included 2,663 subjects who participated in the medical checkup program with baseline CACS measurement between January 2010 and December 2010. Those subjects also underwent repeated medical checkups and CACS measurements between January 2014 and December 2014. Among them, a total of 1,575 subjects were enrolled for the final analysis after exclusion for the following reasons: regression of CAC (n = 45); presence of self-reported history of coronary artery disease (n = 28), ischemic stroke (n = 8), or diabetes (n = 187); missing data (n = 31); alcohol consumption history (≥3 times/week: n = 514); prior history of hepatitis or positive hepatitis B/C viral serologic marker (n = 79) or CACS >0 (n = 214).

The study was approved by the institutional review board of Kangbuk Samsung Hospital. The requirement for obtaining patient informed consent was waived because we used de-identified data that were routinely collected during the health screening process.

### Anthropometric and laboratory measurements

Serum biochemical parameters and physical measurements were obtained by trained staff during health examinations, and data on medical history, medication use, and health-related behaviors were collected through a self-administered questionnaire.

Body mass index (BMI) was calculated by dividing weight (kg) by height (m) squared. Systolic blood pressure (SBP) and diastolic blood pressure were measured three times with the participants in a seated position, with a 1-min rest between each measurement. The average of the second and third measurements was used in our analyses.

The blood samples were collected after 12 hours of overnight fasting. Fasting plasma glucose (FPG) concentrations were determined by the hexokinase method (Modular D2400; Hitachi, Tokyo, Japan). An electrochemiluminescence immunoassay was used to assess fasting insulin concentrations (Modular E170; Hitachi). An enzymatic colorimetric test was used to measure total cholesterol (TC) and triglyceride (TG) concentrations. The selective inhibition method was used to measure high-density lipoprotein cholesterol (HDL-C) levels, and a homogeneous enzymatic calorimetric test was used to measure low-density lipoprotein cholesterol (LDL-C) levels. Alanine aminotransferase (ALT) level was measured by UV without the P5P method (Advia 1650 Autoanalyzer, Bayer Diagnostics, Leverkusen, Germany).

Systemic inflammation was assessed by hs-CRP. Subjects were divided into low and high hs-CRP groups according to median value of hs-CRP in 2010. Serum hs-CRP levels were measured using a nephelometric assay with a BNII nephelometer (Dade Behring, Deerfield, IL). The median value for hs-CRP in 2010 was 0.06 mg/L.

Insulin resistance was measured using the homeostatic model assessment of insulin resistance (HOMA-IR) and was obtained by applying the following formula: HOMA-IR = fasting insulin (μIU/mL) × fasting blood glucose (mmol/L) / 22.5 [18].

A smoker was defined as a subject who had smoked at least five packs of cigarettes over the course of his or her life. Regular exercise was defined as the performance of moderate intensity exercise at least three times every week.

### Assessment of fatty liver by hepatic ultrasonography

Abdominal ultrasonography (US) was used for the evaluation of hepatosteatosis (Logic Q700 MR; GE, Milwaukee, WI, USA). US is the most frequently utilized modality in fatty liver evaluation, with a reported high sensitivity of over 90% and specificity of over 80% [19]. Since hepatic steatosis leads to an increase in the echogenicity of the liver parenchyma, the liver appears brighter than the cortex of the kidney on US [20]. For this study, abdominal ultrasonography (ASPEN; Acuson, PA, USA) using a 3.5 MHz probe was performed in all subjects by experienced clinical radiologists, and fatty liver was diagnosed based on standard criteria, including hepatorenal echo contrast, liver brightness, and vascular blurring.

### Measurements of CACS

CACS was measured by multi-detector computed tomography. All computed tomography scans were obtained with a Lightspeed VCT XTe-64 slice MDCT scanner (GE Healthcare, Tokyo, Japan) with a standardized scanning protocol, using 40 × 2.5-mm section collimation, 400-ms rotation time, 120-kV tube voltage, and 124-mAs (310 mA × 0.4 second) tube current under ECG-gated dose modulation. Quantitative CACS was calculated according to the method described by Agatston et al. [21].

### Statistical analysis

For all of the analyses, the values for ALT, TG, HOMA-IR, and hs-CRP were log-transformed. Continuous variables were expressed as mean ± SD for normally distributed variables or median (interquartile range) for variables not normally distributed. Continuous variables were compared using independent *t*-tests, non-normally distributed variables were compared using Mann-Whitney U tests, and categorical variables were expressed as percentages and compared between groups using the chi test. The study population was categorized into four groups according to NAFLD state and hs-CRP levels as follows: no NAFLD & hs-CRP <0.06 mg/L (n = 515), no NAFLD & hs-CRP ≥0.06 mg/L (n = 326), NAFLD & hs-CRP <0.06 mg/L (n = 261), and NAFLD & hs-CRP ≥0.06 mg/L (n = 473).

Student’s *t*-test and a one-way analysis of variance (ANOVA) were used to assess the differences across each group with continuous variables. Pearson’s chi square test was performed for the assessment of the differences with categorical variables.

Multivariate logistic regression analysis was performed to estimate the odds ratio (OR) and 95% confidence interval (95% CI) of the likelihood of CAC development across study groups. Four models were designated: model 1 was unadjusted; model 2 was adjusted for age and sex; model 3 was adjusted for age, sex, FPG, TC, SBP, and smoking; and model 4 was further adjusted for BMI. A p-value <0.05 was considered statistically significant. To examine the differences in the change of CACS over the groups according to the presence of NAFLD by hs-CRP, a one-way analysis of covariance (ANCOVA) with post-hoc comparisons was used. All statistical analyses were performed with SPSS version 23.

## Results

### Comparison of the baseline characteristics of the participants according to NAFLD status, hs-CRP levels, and CAC development

The baseline biochemical and clinical characteristics of the study subjects divided by the baseline presence of NAFLD and hs-CRP levels (higher and lower half divided by 0.06 mg/L) are shown in Table 1, respectively.

**Table 1:** Comparison of baseline characteristics according to the presence of NAFLD and hs-CRP levels at baseline.

N = 1,575	Overall	No NAFLD (N = 841)	NAFLD (N = 734)	P-value	Hs-CRP <0.06 mg/L (N = 776)	Hs-CRP ≥0.06 mg/L (N = 799)	P-value
Age (years)	39.9±5.4	39.8±5.5	40.0±5.3	0.024	39.9±5.5	39.9±5.4	0.836
Men (%)	1,411 (89.6)	715 (85.0)	696 (94.8)	<0.01	684 (88.1)	727 (91.0)	0.039
BMI (kg/m^2^)	24.6±3.0	23.3±2.5	26.2±2.7	0.01	23.6±2.6	25.6±3.0	<0.01
Proportion of subjects with BMI ≥25 kg/m^2^ (%)	640 (40.6)	173 (20.6)	467 (63.6)	<0.01	211 (27.2)	429 (53.7)	<0.01
SBP (mmHg)	117.9±11.9	115.9±12.2	120.1±11.1	<0.01	116.5±11.8	119.3±11.8	<0.01
Smoking (≥5PY, %)	838 (53.2)	352 (41.9)	385 (52.5)	<0.01	347 (44.7)	390 (48.8)	0.057
FPG (mg/dL)	94.4±8.3	93.3±8.1	95.7±8.4	<0.01	93.6±7.9	95.2±8.6	<0.01
ALT (IU/L)	29.2±19.9	21.5±10.9	38.0±24.0	<0.01	24.8±14.9	33.5±23.1	<0.01
TC (mg/dL)	206.5±35.4	201.0±35.4	212.8±34.4	<0.01	203.0±34.8	210.0±35.7	<0.01
TG (mg/dL)	143.2±89.7	113.0±59.5	177.7±104.7	<0.01	131.0±78.0	155.0±98.3	<0.01
HDL-C (mg/dL)	51.8±12.3	55.7±12.8	47.3±10.0	<0.01	53.8±13.0	49.8±11.3	<0.01
LDL-C (mg/dL)	129.8±32.2	124.4±31.7	136.0±31.6	<0.01	125.9±32.0	133.7±31.8	<0.01
Hs-CRP (mg/L)	0.13±0.4	0.10±0.3	0.16±0.4	<0.01	0.03±0.0	0.22±0.5	<0.01
HOMA-IR	1.4±0.9	1.1±0.6	1.8±1.0	<0.01	1.2±0.7	1.6±1.0	<0.01
CACS change	1.1±5.6	0.8±4.9	1.4±6.3	<0.01	0.7±4.3	1.4±6.7	<0.01

NAFLD, non-alcoholic fatty liver disease; BMI, body mass index; SBP, systolic blood pressure; CACS, coronary artery calcium score; FPG, fasting plasma glucose; ALT, alanine aminotransferase; TC, total cholesterol; TG, triglycerides; HDL-C, high-density lipoprotein cholesterol; LDL-C, low-density lipoprotein cholesterol; hs-CRP, high-sensitivity C-reactive protein; HOMA-IR, homeostasis model assessment of insulin resistance.

**Fig 1:**
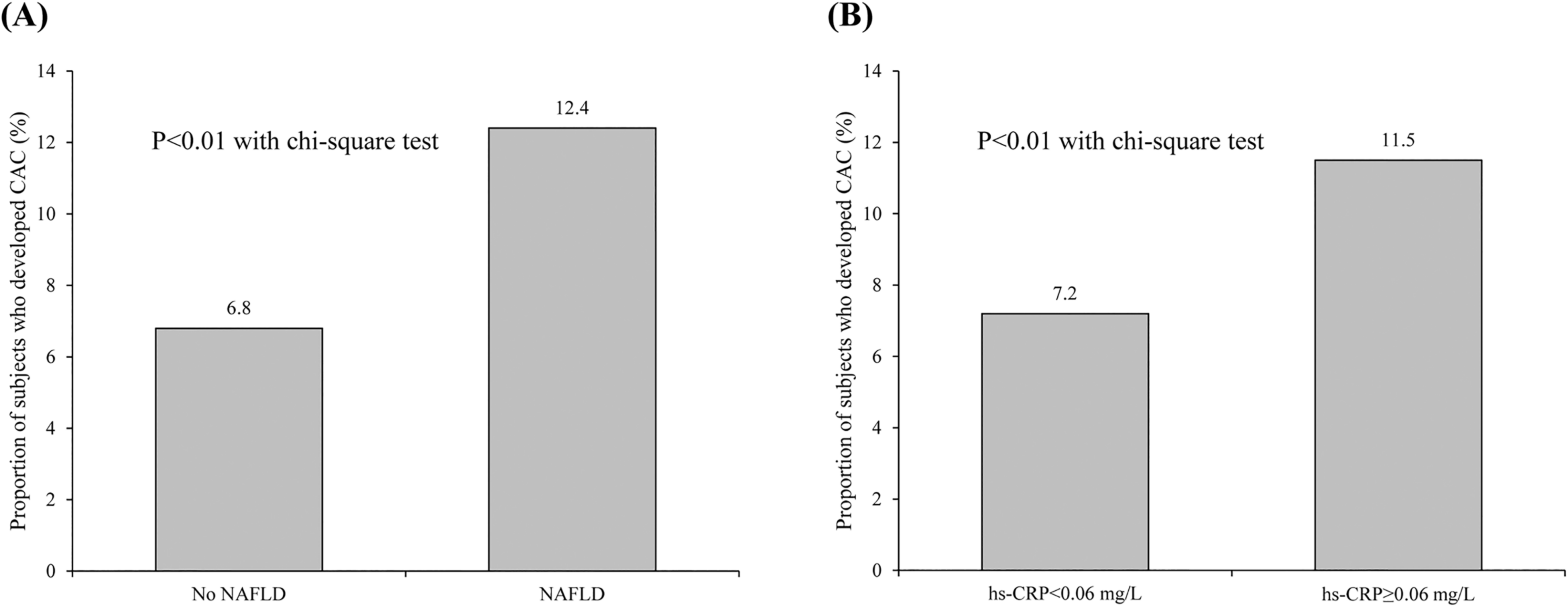
**Proportion of subjects who developed CAC over four years according to baseline NAFLD status (A) and hs-CRP levels (B).** CAC, coronary artery calcification; NAFLD, non-alcoholic fatty liver disease; hs-CRP, high-sensitivity C-reactive protein.

Overall, 734 (46.6%) subjects had NAFLD at baseline. Compared to subjects without NAFLD, those with NAFLD were more likely to be men, obese, and insulin resistant, and to have higher mean values of traditional cardiovascular risk factors including SBP and lipid profiles. The proportion of subjects who developed CAC was significantly higher in subjects with NAFLD at baseline compared with those without NAFLD (12.4 vs. 6.8%, p<0.01), and a greater number of subjects with hs-CRP levels in the higher half of the range developed CAC than those with hs-CRP levels in the lower half (11.5 vs. 7.2%, p<0.01) (Fig 1). The CACS change was higher in the subjects with NAFLD at baseline than in those without NAFLD and this difference was maintained even after controlling for hs-CRP level (Table 1, S1 Table). Moreover, the CACS change was higher in the subjects with higher hs-CRP levels at baseline than in those with lower hs-CRP levels (Table 1).

Over four years, 148 subjects (9.4%) developed CAC. Table 2 shows the baseline characteristics of subjects according to CAC development over four years. The subjects who developed CAC were more likely to be male and smokers and to have higher mean values of BMI, FBS, SBP, hs-CRP, and HOMA-IR, as well as worse lipid profiles.

**Table 2:** Comparison of baseline characteristics according to CAC development over four years.

N = 1,575	No CAC development (n = 1,427, 90.6%)	CAC development (n = 148, 9.4%)	P-value
Age (years)	39.6±5.4	42.3±5.5	<0.01
Male (%)	1,268 (88.9)	143 (96.6)	<0.01
BMI (kg/m^2^)	24.5±3.0	25.7±2.9	<0.01
Proportion of subjects with BMI ≥ 25 kg/m^2^ (%)	556 (39.0)	84 (56.8)	<0.01
SBP (mmHg)	117.5±11.7	121.9±13.5	<0.01
Smoking (≥5PY,%)	655 (45.9)	82 (55.4)	0.017
FPG (mg/dL)	94.1±8.1	97.1±9.7	<0.01
ALT (IU/L)	28.6±19.4	34.8±24.2	<0.01
TC (mg/dL)	205.3±35.4	218.8±32.9	<0.01
TG (mg/dL)	140.6±88.5	167.5±97.5	<0.01
HDL-C (mg/dL)	52.0±12.5	49.0±10.1	<0.01
LDL-C (mg/dL)	128.6±32.1	141.2±30.1	<0.01
Hs-CRP (mg/L)	0.12±0.14	0.13±0.26	0.029
HOMA-IR	0.8±0.3	0.9±0.3	<0.01

CAC, coronary artery calcification; BMI, body mass index; SBP, systolic blood pressure; FPG, fasting plasma glucose; TC, total cholesterol; TG, triglycerides; HDL-C, high-density lipoprotein cholesterol; LDL, low-density lipoprotein cholesterol; hs-CRP, high-sensitivity C-reactive protein; HOMA-IR, homeostasis model assessment model of insulin resistance.

### Comparison of the baseline characteristics of the participants in the four groups divided by NAFLD and hs-CRP level

**Fig 2:**
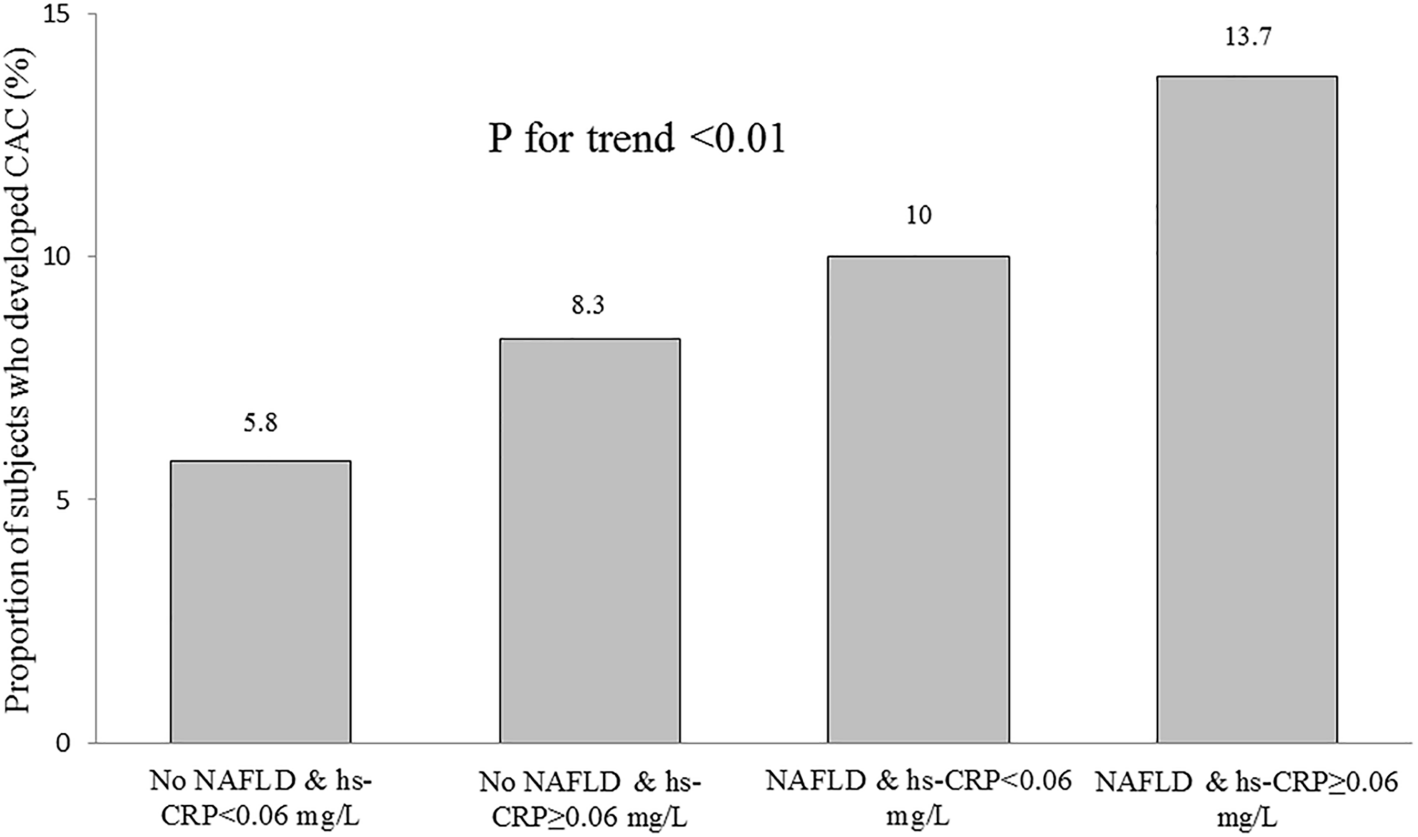
Proportion of subjects who developed CAC over four years according to baseline NAFLD status and hs-CRP levels. CAC, coronary artery calcification; NAFLD, non-alcoholic fatty liver disease; hs-CRP, high-sensitivity C-reactive protein.

Participants were subdivided into four groups according to the presence of baseline NAFLD and serum hs-CRP levels above or below the median value of 0.06 mg/L (Table 3). Subjects with no NAFLD & hs-CRP <0.06 mg/L were the leanest and showed the best metabolic profiles among the four groups. In contrast, subjects with NAFLD & hs-CRP ≥0.06 mg/L showed the highest mean BMI and the worst metabolic profiles among the four groups. Subjects with NAFLD and hs-CRP levels below the median were more obese and showed worse metabolic profiles compared with those without NAFLD and hs-CRP levels above the median (Table 3). The proportion of subjects who developed CAC significantly increased in the order of no NAFLD & hs-CRP <0.06 mg/L, no NAFLD & hs-CRP ≥0.06 mg/L, NAFLD & hs-CRP <0.06 mg/L, and NAFLD & hs-CRP ≥0.06 mg/L (5.8%, 8.3%, 10%, and 13.7%, respectively; p for trend<0.01) (Fig 2).

**Table 3:** Comparison of baseline characteristics according to baseline NAFLD status and hs-CRP levels.

N = 1,575	No NAFLD & hs-CRP <0.06 mg/L (n = 515)	No NAFLD & hs-CRP ≥0.06 mg/L (n = 326)	NAFLD & hs-CRP <0.06 mg/L (n = 261)	NAFLD & hs-CRP ≥0.06 mg/L (n = 473)	P-value
Age (years)	39.6±5.6	40.1±5.4	40.4±5.2	39.8±5.4	0.161
Male (%)	433 (84.1)	282 (86.5)	251 (96.2)	445 (94.1)	<0.01
BMI (kg/m^2^)	22.8±2.3	24.1±2.5	25.3±2.4	26.7±2.8	<0.01
Proportion of subjects with BMI ≥25 kg/m^2^ (%)	75 (14.6)	98 (30.1)	136 (52.1)	331 (70.0)	<0.01
SBP (mmHg)	115.3±12.3	117.0±12.1	118.8±10.4	120.8±11.3	<0.01
Smoking (≥5PY,%)	205 (39.8)	147 (45.1)	142 (54.4)	243 (51.4)	<0.01
FPG (mg/dL)	92.9±7.9	93.8±8.2	94.9±7.7	96.2±8.8	<0.01
ALT (IU/L)	20.7±10.2	22.8±11.8	32.9±19.0	40.8±25.9	<0.01
TC (mg/dL)	200.3±34.7	202.3±36.3	208.3±34.2	215.3±34.3	<0.01
TG (mg/dL)	109.2±54.7	119.0±66.1	173.8±97.1	179.9±108.7	<0.01
HDL-C (mg/dL)	56.9±13.2	53.8±11.8	47.8±10.1	47.0±10.0	<0.01
LDL-C (mg/dL)	123.2±32.2	126.4±30.7	131.3±31.0	138.6±31.7	<0.01
Hs-CRP (mg/L)	0.03±0.01	0.20±0.45	0.04±0.01	0.23±0.51	<0.01
HOMA-IR	1.0±0.6	1.2±0.6	1.6±0.9	1.9±1.1	<0.01

NAFLD, non-alcoholic fatty liver disease; hs-CRP, high-sensitivity C-reactive protein; BMI, body mass index; SBP, systolic blood pressure; CACS, coronary artery calcium score; FPG, fasting blood glucose; ALT, alanine aminotransferase; TC, total cholesterol; TG, triglycerides; HDL-C, high-density lipoprotein cholesterol; LDL-C, low-density lipoprotein cholesterol; HOMA-IR, homeostasis model assessment of insulin resistance.

### Risk of CAC development after four years according to baseline NAFLD and hs-CRP levels

The risk for CAC development was 1.8-fold greater in subjects with baseline NAFLD compared to those without baseline NAFLD (OR 1.80; 95% CI 1.26–2.56).

When the risk was assessed in the four groups divided by baseline NAFLD status and hs-CRP level above or below the median of 0.06 mg/L, the OR for CAC development was highest in the subjects with NAFLD & hs-CRP ≥0.06 mg/L after adjustment for age, sex, ALT, FPG, TC, SBP, LDL-C and smoking (model 3, Table 4). However, this significant increase in OR also disappeared when BMI was included in the model (model 4, Table 4).

**Table 4:** Relative risk for CAC development according to baseline NAFLD and hs-CRP levels.

	CAC progression	
	No NAFLD	NAFLD
	OR (95% CI)	OR (95% CI
Model 1		
overall	1	1.80 (1.26–2.56)
hs-CRP <0.06	1	1.53 (0.88–2.67)
hs-CRP ≥0.06	1.39 (0.80–2.40)	2.40 (1.52–3.81)
Model 2		
overall	1	1.43 (0.97–2.11)
hs-CRP <0.06	1	1.28 (0.72–2.27)
hs-CRP ≥0.06	1.33 (0.77–2.30)	1.87 (1.13–3.09)
Model 3		
overall	1	1.33 (0.90–1.97)
hs-CRP <0.06	1	1.22 (0.68–2.17)
hs-CRP ≥0.06	1.28 (0.73–2.22)	1.67 (1.01–2.77)
Model 4		
overall	1	1.23 (0.82–1.83)
hs-CRP <0.06	1	1.08 (0.60–1.94)
hs-CRP ≥0.06	1.18 (0.68–2.07)	1.37 (0.79–2.35)

CAC, coronary artery calcification; NAFLD, non-alcoholic fatty liver disease; hs-CRP, high-sensitivity C-reactive protein; FBS, fasting blood sugar; TC, total cholesterol; SBP, systolic blood pressure; BMI, body mass index; ALT, alanine aminotransferase

Model 1 was adjusted for age and sex; model 2 was adjusted for age, sex, ALT, and smoking; model 3 was adjusted for age, sex, ALT, smoking, FBS, and LDL; and model 4 was adjusted for the variables in model 3 plus BMI.

## Discussion

In our four-year, retrospective cohort study, we observed that a greater number of subjects with NAFLD developed CAC. In addition, we demonstrated that subjects with concomitant baseline NAFLD and systemic inflammation as determined by hs-CRP levels above the median showed a significantly higher risk for CAC development compared with the other groups. However, this significance was attenuated after adjusting for BMI, which reveals the importance of obesity in the relationship between NAFLD, systemic inflammation, and subclinical atherosclerosis.

Recent studies have suggested that the presence of NAFLD is a cardiovascular risk factor, since many clinical studies reported increased CVD in patients with NAFLD compared with those without NAFLD [7,22,23]. Since NAFLD is strongly linked to insulin resistance, which is the main pathogenic mechanism underlying diabetes and metabolic syndrome, the association between NAFLD and metabolic diseases is expected. The presence of NAFLD in a subject implies the presence of numerous abnormalities related to visceral obesity and the accumulation of ectopic fat, since NAFLD is one of the manifestations of dysfunctional adipose tissue [24]. Therefore, the risk for subclinical atherosclerosis could be higher in subjects with NAFLD.

The association between NAFLD and CAC has previously been demonstrated [17,25]. However, in contrast to prior findings, our present data indicate that this association is attenuated after accounting for BMI, which is accordance with the findings of the CARDIA study [26]. The difference in results can be attributed to the fact that the mean age of our study population was over 10 years younger than that in the other studies. Indeed, Jason et al. [27] showed that BMI presents an inverse relationship with coronary artery calcification in old age. The mean age of our study population was in the early 40s, and decreases in BMI are rare at that age. Therefore, our findings suggest that obesity assessed by BMI is likely to be a key mediator of the connection between NAFLD, greater systemic inflammation, and the development of CVD in young subjects.

Interestingly, we found that the risk of CAC development differed according to level of systemic inflammation, which is a known mediator or biomarker of adverse outcomes in individuals with NAFLD [6,28]. Subjects with NAFLD and higher hs-CRP levels had the highest risk for development of CAC. Interestingly, subjects with NAFLD but lower hs-CRP levels had a higher prevalence of CAC development over four years compared to those without NAFLD but with higher hs-CRP levels (10 vs. 8.3%), suggesting that the presence of NAFLD is more influential in the development of CAC than is higher systemic inflammation as assessed by hs-CRP. Although the simultaneous existence of NAFLD and systemic inflammation significantly increased the risk for CAC development, only the presence of NAFLD was shown to affect CAC development. These results suggest that not only must NAFLD be prevented in our patients through lifestyle modification and weight loss, but also other metabolic risk factors that could affect systemic inflammation should be considered in order to avoid subclinical atherosclerosis. In other words, our results imply that special attention should be paid to young, relatively healthy subjects with NAFLD and higher systemic inflammation.

Our study has several limitations. First, fatty liver was diagnosed via liver ultrasonography, but this equipment is well known to have rather limited sensitivity, and liver fat infiltration of less than 30% of the total liver weight can be hard to detect [29]. This limitation may be justified by the fact that this sonography-guided diagnostic modality is common for fatty liver and is not a unique practice applied only to our study [14,30–32]. Ultrasonography was performed by experienced clinical radiologists in accordance with the four known clinical diagnostic criteria of NAFLD: hepatorenal echo contrast, liver brightness, deep attenuation, and vascular blurring. Furthermore, it would be inappropriate to perform invasive liver biopsies in a large-scale epidemiologic study. The significant predominance of males in the study population is another factor to be considered. Moreover, our study population was limited to a single ethnic group. The impact of hs-CRP on CAC development in NAFLD individuals should be further evaluated in young, healthy, and multi-ethnic populations. Third, the retrospective design of the current study might have allowed unknown confounder factors to affect the observations. Despite these limitations, this is the first study to show that systemic inflammation might be a crucial factor in CAC development in individuals with NAFLD in an Asian population. Additionally, by targeting a relatively young, healthy, and large group of subjects from health check-up programs and excluding previously diagnosed coronary artery disease, stroke, and/or diabetes, we provided significant evidence of the relationship between NAFLD and CAC development.

In conclusion, the risk of CAC development was significantly higher in patients with NAFLD and higher levels of systemic inflammation than in those without NAFLD and with low levels of systemic inflammation in our study of relatively healthy young subjects. Therefore, in asymptomatic subjects with NAFLD, especially those with high hs-CRP, prevention of subclinical atherosclerosis through lifestyle modification and further reversal of fatty liver and systemic inflammation is critical.

## Supporting information

S1 Table Hs-CRP-adjusted means (± standard error) of the CAC score change and BMI between the no NALFD and NAFLD groups. (DOCX)
