# Supplementary material for: Increased risk for development of coronary artery calcification in subjects with non-alcoholic fatty liver disease and systemic inflammation
Source: PLoS One. 2017 Jul 7;12(7):e0180118. doi: 10.1371/journal.pone.0180118 (PMC5501459; doi:10.1371/journal.pone.0180118)
Supplement: S1 Table — (DOCX) [file pone.0180118.s001.docx]

**S1 Table.** Hs-CRP-adjusted means (± standard error) of the CAC score change and BMI between the no NALFD and NAFLD groups

|  | No NAFLD (N=841) | NAFLD (N=734) | P-value |
| --- | --- | --- | --- |
| hs-CRP | 0.10±0.29 | 0.16±0.42 | 0.002 |
| CAC change | 0.14±0.02 | 0.25±0.02 | 0.005 |
| BMI | 23.31±0.09 | 26.18±0.10 | <0.001 |

NAFLD, non-alcoholic fatty liver disease; hs-CRP, high-sensitivity C-reactive protein; CAC, coronary artery calcification; BMI, body mass index.
